# Clinical Evidence in Respiratory and Urinary Tract Infections: Focus on Ceftibuten

**DOI:** 10.1159/pha/aejag003

**Published:** 2026-08-31

**Authors:** Gabriele Cortellini, Marzia Del Re, Eduardo Ponticiello, Matteo Bassetti

**Affiliations:** a Allergy Unit, Azienda Sanitaria della Romagna, Rimini, Italy; b Saint Camillus International University of Medical and Health Sciences, Rome, Italy; c Fondazione IRCCS Policlinico A. Gemelli, Rome, Italy; d Pediatric Unit AORN San Giuseppe Moscati Avellino, Rome, Italy; e Università degli Studi di Genova, IRCCS Ospedale Policlinico San Martino, Genova, Italy

**Keywords:** Antibiotic, Antibiotic resistance, Ceftibuten

## Abstract

**Background:**

Antimicrobial resistance (AMR) is one of the most pressing global health problems, resulting from the misuse and overuse of antibiotics across various sectors, which has led to the emergence and spread of resistant microorganisms. AMR occurs when microorganisms no longer respond to antibiotics. In Italy alone, AMR is responsible for over 10,000 deaths each year, exceeding 40% of all AMR, attributable deaths in the EU/EEA. This alarming trend highlights the urgent need to preserve effective treatment options and to re-evaluate the role of well-established oral antibiotics with a favourable pharmacological and microbiological profile.

**Summary:**

Ceftibuten is an oral third-generation cephalosporin with potent activity against many Gram-negative and some Gram-positive pathogens, including several Gram-negative pathogens. It is approved for the treatment of respiratory and urinary tract infections (UTIs), both uncomplicated and complicated, in adults as well as in children. This review discusses clinical and pharmacological data supporting ceftibuten’s efficacy, safety, pharmacokinetics, and microbiological spectrum. Moreover, the paper discusses its potential role in contemporary clinical practice, especially considering recent supply interruptions and increasing resistance patterns.

**Key Messages:**

Ceftibuten represents an oral treatment option for respiratory and UTIs, particularly where resistance to other agents limits therapeutic choices. Its favourable pharmacokinetic profile, safety both in paediatric and adult populations, and efficacy against key resistant pathogens support. use within approved indications. Finally, this antibiotic offers a favourable safety profile for both patients allergic to penicillin and cephalosporins, particularly third- and fourth-generation cephalosporins. Ensuring continued access to this antibiotic may contribute to appropriate management of infections in both community and hospital settings.

## Introduction

Antimicrobial drugs represent a broad class of pharmacological agents used to treat infections caused by a wide variety of pathogens. They include antibiotics (for bacterial infections), antivirals, antifungals, and antiparasitic such as antimalarials [1]. Since their introduction in the early 20th century, these agents have saved millions of lives by controlling infections and enabling high-risk medical procedures, such as surgery, organ transplantation, dialysis, and chemotherapy [2]. Without reliable antimicrobial therapy, these interventions would carry a significantly higher risk of severe infections including sepsis, morbidity, and mortality [1].

Antimicrobial resistance (AMR) is a natural evolutionary process with resistance genes identified in ancient bacterial genomes [3]. AMR arises when microorganisms, particularly bacteria, evolve to resist the effects of antimicrobial agents, leading to the emergence of “superbugs” such as MRSA (methicillin-resistant *Staphylococcus aureus*) and extensively drug-resistant tuberculosis, that are increasingly difficult to treat [2]. Notably, six bacterial pathogens (*Escherichia coli*, *Staphylococcus aureus*, *Klebsiella pneumoniae*, *Streptococcus pneumoniae*, *Acinetobacter baumannii*, and *Pseudomonas aeruginosa*) are responsible for over 73% of all AMR-related deaths (95% uncertainty interval: 66.9–78.8) and seven pathogen-drug combinations were responsible for more than 50,000 deaths each, including methicillin-resistant *S. aureus*, third-generation cephalosporin-resistant *E. coli* and *K. pneumoniae*, fluoroquinolone-resistant *E. coli*, carbapenem-resistant *K. pneumoniae* and *A. baumannii*, and macrolide-resistant *Streptococcus pneumoniae* [4].

According to recent estimates from the Global Burden of Disease (GBD) 2021 study, bacterial AMR was associated with approximately 4.71 million deaths (95% uncertainty interval: 4.23–5.19 million) in 2021, whereas 1.14 million were directly attributable to resistant infections [5]. In Europe, AMR is responsible for approximately 35,000 deaths annually. According to the WHO and ECDC, one-third of all infections are now caused by resistant bacteria, with up to 70–75% of these cases occurring in healthcare settings [6]. AMR also represents a growing challenge in the outpatient setting, where most community-acquired respiratory and urinary tract infections (UTIs) are managed, and the availability of effective oral antibiotics remains essential for appropriate antimicrobial stewardship.

In Italy, antibiotic resistance represents one of the most severe public health threats. The mortality rate attributable to AMR is among the highest in Europe. The national burden is estimated at over 10,000 deaths per year, accounting for more than 40% of the total reported in the EU/EEA [6]. Projections suggest by 2050, AMR-related mortality could reach 10 million globally per year [4]. Efforts to combat AMR must include enhanced infection prevention and control, improved access to second-line antimicrobials, development of vaccines and novel antibiotic agents [4].

Ceftibuten, originally approved in 1995, is a semisynthetic, β-lactamase-stable, third-generation cephalosporin with antibacterial activity [7]. Ceftibuten is active against *Enterobacterales* and stable against many class A β-lactamases produced by these organisms [8]. Therapeutic indications include the treatment of both upper and lower respiratory tract and urinary infections, especially those acquired in the community [7]. Ceftibuten’s favourable pharmacokinetic profile, rapid bactericidal action at or slightly above MIC levels, and reduced allergenic potential compared to other cephalosporins, support its use within approved indications [8]. Given its oral formulation and favourable pharmacokinetic profile, ceftibuten may represent a useful option in the outpatient management of selected community-acquired infections.

## Current Approved Indications and Guideline Recommendations

According to the Summary of Product Characteristics [7], ceftibuten is approved for the treatment of upper respiratory tract infections (pharyngitis, tonsillitis, sinusitis, otitis media), lower respiratory tract infections (acute exacerbations of chronic bronchitis), and uncomplicated and complicated UTIs. Although ceftibuten is not specifically mentioned in most contemporary international guidelines, its approved indications remain consistent with current therapeutic strategies for common community-acquired infections [9, 10].

The European Association of Urology (EAU) 2024 guidelines include oral third-generation cephalosporins among the therapeutic options for selected patients with uncomplicated pyelonephritis [11], although individual agents are not specified. Likewise, current respiratory guidelines preferentially recommend other oral antibiotics as first-line empirical therapy, without specifically addressing ceftibuten [9, 10]. Consequently, the absence of explicit recommendations should not be interpreted as evidence against its clinical value but rather reflects the evolution of guideline development and the tendency to recommend antibiotic classes rather than individual agents [9–11].

Ceftibuten retains several pharmacological characteristics that continue to support its clinical usefulness, including potent activity against many *Enterobacterales* and common respiratory pathogens, excellent oral bioavailability, prolonged half-life allowing once-daily administration, favourable tolerability, and a low propensity for clinically relevant β-lactam cross-reactivity. These properties make it a valuable oral therapeutic option, particularly for UTIs and selected respiratory infections, as well as for patients requiring alternative β-lactam therapy because of documented allergy or intolerance.

The clinical importance of maintaining ceftibuten among available oral antibiotics has recently been highlighted in Italy. In September 2023, the Italian Association of Territorial and Hospital Allergists and Immunologists (AAIITO) formally requested Recordati to reconsider the discontinuation of ceftibuten, emphasizing its favourable safety profile, particularly for patients with β-lactam allergy. The request was subsequently submitted to the Italian Medicines Agency (AIFA), together with the supporting scientific documentation.

Within this framework, a multidisciplinary panel of Italian experts critically reviewed the available evidence regarding the efficacy and safety of ceftibuten in upper and lower respiratory tract infections and UTIs. The review considered the clinical literature together with the pharmacokinetic, microbiological, and pharmacodynamic characteristics of ceftibuten as an orally active third-generation cephalosporin. Particular attention was also devoted to its allergological profile, recognizing the need to preserve the availability of an oral cephalosporin with limited cross-reactivity within the β-lactam class for selected patients in routine clinical practice.

## Discussion

### Pharmacokinetics

Ceftibuten exhibits dose-dependent pharmacokinetics, with modest accumulation after multiple daily doses of 400, 600, or 800 mg [12]. It is rapidly absorbed via the oral route, with an oral bioavailability ranging between 75% and 90% and a peak plasma concentration of approximately 17 mg/L following a standard adult dose of 400 mg [12]. These pharmacokinetic characteristics are associated with measurable drug concentrations in several tissues.

Absorption primarily occurs via aqueous channel filtration and active transport, notably involving the PEPT1 transporter [13]. A recent pharmacokinetic review confirmed that ceftibuten achieves measurable concentrations in key biological fluids and tissues, including plasma, tonsils, bronchial mucosa, and urine [13]. Furthermore, tissue elimination is generally slower than plasma clearance, resulting in more prolonged concentrations in tissues compared with plasma [14]. In paediatric patients aged 6 months to 17 years, a once-daily dose of 9 mg/kg results in plasma concentrations within the reported therapeutic range. Peak levels of 12–16 μg/mL were consistent across all paediatric age groups, demonstrating age-independent pharmacokinetic behaviour [15]. These findings have been reinforced by recent population PK models confirming the predictability of exposure and consistency with once-daily dosing in both children and adults [13].

Ceftibuten demonstrates a half-life of approximately 2–3 h and undergoes minimal hepatic metabolism, producing trans-ceftibuten, a metabolite with approximately eightfold lower antimicrobial activity than the parent drug. Following oral administration, approximately 60–64% of the absorbed dose is bound to plasma proteins, and the apparent volume of distribution is around 0.2 L/kg, suggesting distribution in the interstitial fluid compartment [12, 13].

Renal excretion is the major elimination route, with 60–70% of the administered dose recovered in the urine as active cis-ceftibuten, and an additional 10–20% as trans-ceftibuten. Patients with renal impairment may exhibit reduced clearance, resulting in increased systemic exposure (AUC), requiring dose adjustment in this population. In patients with creatinine clearance of 30–49 mL/min, the daily dose should be reduced by half, while further dose adjustment is recommended when creatinine clearance is < 30 mL/min [7, 12, 13].

### Mechanism of Action

Ceftibuten exerts its antibacterial activity by binding to and inactivating penicillin-binding proteins (PBPs), which are located on the inner membrane of the bacterial cell wall. These enzymes play a crucial role in the final stages of peptidoglycan synthesis and in remodelling the cell wall during bacterial growth and division. Inactivation occurs through the formation of a stable covalent bond between ceftibuten and a serine residue in the active site of the PBPs. This disrupts the cross-linking of peptidoglycan chains, compromising the structural integrity of the bacterial cell wall and ultimately leading to cell lysis. Ceftibuten exhibits a particular affinity for PBP3, a transpeptidase essential for septal formation during bacterial division.

The inhibition of this enzyme effectively halts cell division and is especially relevant for its bactericidal activity against Gram-negative organisms. It also shows moderate binding affinity to PBPs 1a and 1b, which are involved in maintaining the cell wall’s mechanical strength [16]. In addition to direct antibacterial effects, binding of ceftibuten to PBPs may induce morphological changes that increase bacterial susceptibility to phagocytosis and reduce bacterial adhesion to host epithelial surfaces [15].

### Clinical Efficacy of Ceftibuten

Ceftibuten has demonstrated clinical efficacy in treating upper respiratory tract infections (pharyngitis, tonsillitis, sinusitis, and otitis media), lower respiratory tract infections (bronchitis, pneumonia, and bronchopneumonia), and UTIs (cystopyelitis, cystitis, urethritis) [7]. In patients with otitis media, ceftibuten has been shown to penetrate middle ear fluid, achieving concentrations comparable to plasma levels following oral administration [17]. Ceftibuten reaches the middle ear fluids rapidly; at 4 h after its administration, its concentration overlaps with plasma levels and is detectable up to 12 h post-oral administration. In the treatment of paediatric otitis media, a single 5-day therapeutic cycle of 9 mg/kg/day has been shown to be significantly as effective as a 10-day therapeutic cycle. The shorter treatment duration and once-daily dosing schedule may facilitate adherence in paediatric patients [17, 18]. Ceftibuten has been evaluated in the treatment of acute sinusitis. In fact, this antibiotic has been indicated, since 2013, as a valid and effective therapeutic option in this context within the “Document on the appropriate use of antibiotics”, particularly for paediatric patients, from the Sicilian Regional Center for the Coordination of Pharmacovigilance (Health Department) [19].

In the review by Wiseman et al., clinical responses after treatment for bacterial sinusitis with once-daily ceftibuten in both adults and children occurred in all patients (with clinical recovery in 68%); the response rate was found to be similar to that of patients treated with amoxicillin/clavulanic acid 500 mg three times daily (TID) [20]. In cases of pharyngitis, treatment with ceftibuten at a dose of 9 mg/kg/day administered once daily was associated with higher clinical and bacteriological response rates compared with phenoxymethylpenicillin 25 mg/kg/day (3 fractionated doses) in children and adolescents (aged 3–18 years) with symptomatic pharyngitis or scarlet fever caused by group A β-haemolytic streptococcus [20]. Five to 7 days after treatment, clinical response was documented in 97% of patients treated with ceftibuten compared to 89% of patients treated with phenoxymethylpenicillin (*p* < 0.01) and the bacterial elimination rate was 91% vs. 80% for the two groups respectively (*p* < 0.01). This elimination rate remained higher in the ceftibuten group (89% vs. 79%) at 2–3 weeks after treatment, with no significant difference between the two groups [20].

Ceftibuten is also used as an oral treatment option in UTIs. Recurrent UTIs are defined as at least three episodes per year or two within 6 months. They may involve either the lower (cystitis) or upper urinary tract (pyelonephritis), although repeated episodes of pyelonephritis should raise suspicion of an underlying complicated cause.

UTIs are associated with a significant decline in quality of life, affecting social interactions, sexual wellbeing, self-esteem, and occupational functioning [11]. Based on the 2024 European Association of Urology (EAU) Guidelines, oral fluoroquinolones and cephalosporins are among the recommended options for empiric therapy of uncomplicated pyelonephritis.

However, the guidelines also emphasize that oral administration of cephalosporins results in lower systemic and urinary drug concentrations compared to intravenous formulations, which may impact clinical efficacy in certain scenarios. For the treatment of UTIs, ceftibuten is indicated at a dosage of 400 mg once daily, with treatment duration ranging from 5 to 10 days, depending on the infection’s severity and site of involvement [11]. Ceftibuten has demonstrated clinical efficacy in the treatment of UTIs in several clinical trials [21–23].

Ceftibuten has demonstrated clinical efficacy in the treatment of febrile UTIs in paediatric patients. A randomized multicentre study published in Paediatric Nephrology evaluated ceftibuten in children aged 1 month to 12 years with febrile UTIs, reporting a higher cure rate compared with TMP-SMX (93% vs. 83% after 10 days of treatment).

On the other hand, a study conducted on UTIs in adults, using susceptibility testing on *E. coli* isolates, reported a susceptibility rate of 99.4% for ceftibuten, compared with ciprofloxacin (21%), cephalexin (41%), and TMP/SMX (31.7%) [24]. Wiseman et al. [20] report 93%–100% of adult patients treated with ceftibuten 100 or 200 mg twice daily (BID) or 400 mg once daily (QD) achieve good or excellent clinical responses in the treatment of acute uncomplicated UTIs. This result drops to 65–83% in complicated or recurrent infections.

The efficacy of ceftibuten, administered at 100 or 200 mg two or three times daily, has also been investigated in patients with obstetric and gynaecological infections. These include intrauterine and pelvic infections, adnexitis, endometritis, external genital infections, and Bartholin’s abscess, as reported in small non-comparative studies and in a multicentre comparative trial involving 187 patients treated with bacampicillin 250 mg four times daily [25–27].

In two small non-comparative studies involving a total of 20 patients, ceftibuten was associated with clinical responses in cases of acute or chronic bacterial prostatitis. Of course, there are microorganisms resistant to ceftibuten, for example in prostate infections, including Stenotrophomonas (Xanthomonas) maltophilia and *Enterococcus faecalis*. It is reasonable to expect future efficacy from drugs combining ceftibuten with a specific inhibitor, such as ceftibuten-avibactam and ceftibuten-ledaborbactam etzadroxil. It is also reasonable to anticipate their safe future use in terms of allergic reactions [28, 29]. The main causative pathogens, *E. coli*, *Streptococcus* spp., *Staphylococcus* spp., *E. faecalis, and Xanthomonas maltophilia*, were eradicated in 40–50% of patients treated with ceftibuten 200 mg, two or three times daily, for treatment durations ranging from 7 to 28 days [20]. Ceftibuten is currently being evaluated in combination with the novel β-lactamase inhibitors avibactam and ledaborbactam etzadroxil [30–32] for the treatment of complicated UTIs, including acute pyelonephritis. β-lactamases are enzymes involved in bacterial resistance to β-lactam antibiotics. By inhibiting these enzymes, the combination aims to restore the antibacterial activity of ceftibuten against resistant strains. In both cases, ceftibuten has demonstrated in vitro and in vivo activity against β-lactamase-producing Enterobacterales, including strains resistant to current oral and parenteral agents. Table 1 is a summary of key clinical studies on ceftibuten according to the PICO framework.

**Table 1.** Summary of key clinical studies on ceftibuten according to the PICO framework

Study	Population	Intervention	Comparator	Outcomes	Key Findings	Limitations
Wiseman et al., 1994 [20]	Adults with UTI	Ceftibuten 100–400 mg	Various	Clinical response	93–100% efficacy (uncomplicated)	Old studies
Stein et al., 1991 [21]	Adults with uncomplicated UTI	Ceftibuten	None	Clinical cure	High efficacy rates	Small sample
Blumer et al., 1995 [18]	Children with otitis media	Ceftibuten	Cefaclor	Clinical response	Comparable efficacy	Outdated comparator
Kammer RB, Ress R., 1991 [33]	Adults with LRTI	Ceftibuten	Cefaclor	Clinical response	Similar efficacy	Viral aetiology not excluded

### General Tolerability and Safety of Ceftibuten

Ceftibuten, like other β-lactams, is generally well tolerated, with adverse events mostly mild to moderate, primarily involving gastrointestinal discomfort or hypersensitivity reactions. Its high oral bioavailability (75–90%) may limit disruption of intestinal microbial balance, as the small unabsorbed portion is further reduced by anaerobic flora such as Bacteroides fragilis [13, 20]. Early studies in healthy volunteers and animal models showed no significant disruption of the gut microbiota following ceftibuten administration [34]. Another study in healthy volunteers confirmed good absorption and very low faecal excretion of ceftibuten, with transient increase in enterococci and β-lactamase activity in faecal samples during treatment [35].

However, these findings were obtained using older microbiological methods, and more recent approaches based on molecular techniques would be required to better characterize the impact on the gut microbiota. The evaluation of β-lactam antibiotic allergy represents an essential component of antimicrobial stewardship. The removal of inaccurate β-lactam allergy labels helps avoid the unnecessary use of broad-spectrum and costly antibiotics, such as carbapenems [36]. American and European guidelines therefore support risk stratification strategies in patients reporting β-lactam allergy in order to guide an appropriate diagnostic approach, whose costs are largely offset by the clinical and economic benefits for both patients and healthcare systems [37]. A relevant challenge in the collaboration between infectious disease specialists and allergologists is the coexistence of allergy to both penicillins and cephalosporins. This may occur in patients allergic to first-generation cephalosporins or aminocephalosporins, with reported rates ranging from approximately 15%–30% of cases, but it may also involve third- and fourth-generation cephalosporins [20]. For this reason, the availability of cephalosporins with different structural characteristics is particularly important in order to avoid the use of less appropriate β-lactam options, such as carbapenems or non-β-lactam antibiotics that may be more costly or associated with greater toxicity (e.g., fluoroquinolones, vancomycin, teicoplanin, linezolid). Although *E. faecalis* and *Stenotrophomonas maltophilia* were reported among isolates in these studies, both organisms are generally considered intrinsically resistant to ceftibuten and therefore do not represent appropriate therapeutic targets [16]. The development of combinations such as ceftibuten-avibactam and ceftibuten-ledaborbactam may significantly expand its clinical utility [30].

Most third- and fourth-generation cephalosporins share a similar R1 side chain, typically characterized by a methoxyimino group, which is associated with an increased risk of allergic cross-reactivity [38]. Although some molecules, such as ceftazidime, show partial structural differences, these cases are relatively limited and the risk of cross-reactivity may still be clinically relevant [38]. Notable exceptions include the injectable cephalosporins cefazolin [8] and oral ceftibuten. For both drugs, no structural similarity has been demonstrated in the R1 side chain, which represents the main determinant of allergic cross-reactivity. This structural characteristic makes these antibiotics particularly useful therapeutic options in patients with a history of allergy to cephalosporins or concomitant allergy to penicillins [38].

Ceftibuten is also associated with a lower risk of IgE-mediated allergic cross-reactions compared to other penicillins and cephalosporins. This characteristic may make ceftibuten a potential option in selected patients with allergies to aminopenicillins or first-generation cephalosporins, helping avoid the use of more toxic or expensive drugs [20, 38]. Finally, the presence of the carboxy-ethylidene group protects ceftibuten’s β-lactam ring from hydrolysis by many β-lactamases, including several plasmid-mediated β-lactamases, making it more stable than some third-generation parenteral cephalosporins [16, 20, 39].

### Potential Clinical Scenarios beyond Approved Indications

Despite limited formal approval in certain settings, emerging microbiological and clinical data suggest that ceftibuten may have a role in additional scenarios. In particular, its high oral bioavailability and activity against *Enterobacterales* support its potential use as oral step-down therapy after intravenous treatment in systemic Gram-negative infections [40], targeted therapy for infections caused by ESBL-producing organisms (when susceptibility is confirmed), treatment option in outpatient management of complicated UTIs. Recent in vitro studies have demonstrated significant activity of ceftibuten against multidrug-resistant *Enterobacterales*, including ESBL-producing and carbapenem-resistant strains, supporting its potential repositioning in the era of AMR [30]. However, these applications remain limited by the lack of robust phase III randomized controlled trials [41].

## Conclusion

The global rise in AMR has renewed attention on the rational use of oral antibiotics with a well-established efficacy and safety profile. Among these, ceftibuten may represent a useful oral treatment option particularly in the treatment of respiratory and UTIs, two areas heavily affected by resistant Gram-negative pathogens.

Ceftibuten’s pharmacokinetic and pharmacodynamic profile, characterized by high oral bioavailability, stability against several β-lactamases, and selective activity against key pathogens, makes it a potential option for both paediatric and adult populations. Its clinical efficacy has been demonstrated in a range of infections, including otitis media, sinusitis, bronchitis, pneumonia, and various forms of UTIs. Both clinical trials and real-world evidence support its effectiveness in uncomplicated and complicated UTIs, particularly against *E. coli*, the most common uropathogen. In addition, ceftibuten may be considered in selected patients with reported penicillin allergy as well as those with hypersensitivity to aminopenicillins and to third- and fourth-generation cephalosporins, due to its reduced cross-reactivity within the β-lactam class. Ongoing studies exploring its combination with novel β-lactamase inhibitors suggest a promising role for ceftibuten in managing infections caused by resistant organisms.

Following a formal request by the Association of Italian Territorial and Hospital Immunologist Allergologists (AAIITO), which was subsequently submitted to AIFA, the planned withdrawal of ceftibuten from the Italian market was suspended, allowing for its continued clinical use and reinforcing its relevance in current therapeutic practice. Given these characteristics, and the growing need for effective oral alternatives, ceftibuten may represent a valuable option in settings where oral step-down or targeted therapy is appropriate.

## Acknowledgments

The authors acknowledge the editorial assistance provided by Momento Medico S.r.l., Italy.

## Conflict of Interest Statement

Matteo Bassetti receives fees for advisory board, speaking engagements, and research funding from Advanz, Cidara, Gilead, Menarini, MSD, Pfizer, Shionogi, and Mundipharma. Gabriele Cortellini receives fees from Recordati. Maria Del Re receives fees from AstraZeneca, Novartis, Pfizer, Bio-Rad, Johnson & Johnson, Roche, BMS, Daiichi Sankyo, Regeneron, Recordati, QIAGEN, MSD, Lilly, and Menarini. Eduardo Ponticiello declares no conflict of interest.

## Funding Sources

This review was supported by an independent grant from Recordati, dedicated to manuscript writing support and Open Access publication fees. Recordati had no role in study design, execution and analysis, and manuscript conception, planning, writing, and decision to publish.

## Author Contributions

GC and MB contributed to the study conception and to the initial drafting and revision of the manuscript. MDR performed the literature search and data verification and contributed to the critical revision of the content. EP participated in the manuscript revision and in the interpretation of the data. The manuscript was reviewed and approved by GC, MDR, EP, and MB. All authors read and approved the final version of the manuscript and take full responsibility for the integrity of the work.
